# The Lipid-Sensor Candidates CD36 and GPR120 Are Differentially Regulated by Dietary Lipids in Mouse Taste Buds: Impact on Spontaneous Fat Preference

**DOI:** 10.1371/journal.pone.0024014

**Published:** 2011-08-25

**Authors:** Céline Martin, Patricia Passilly-Degrace, Dany Gaillard, Jean-François Merlin, Michaël Chevrot, Philippe Besnard

**Affiliations:** Physiologie de la Nutrition, INSERM U866, Université de Bourgogne, AgroSup Dijon, Dijon, France; Institut Pluridisciplinaire Hubert Curien, France

## Abstract

**Background:**

Recent studies in rodents and humans suggest that the chemoreception of long-chain fatty acids (LCFA) in oral cavity is involved in the spontaneous preference for fatty foods and might contribute to the obesity risk. CD36 and GPR120 are LCFA receptors identified in rodent taste bud cells. The fact that *CD36* or *GPR120* gene inactivation leads to a decrease in the preference for lipids raises the question of the respective role(s) played by these gustatory lipid-sensor candidates.

**Methodology/Principal Findings:**

Using a combination of biochemical, nutritional and behavioural studies in wild-type, *CD36^+/−^*and *CD36^−/−^* mice, it was found that: 1°) CD36 and GPR120 display different diurnal rhythms in the gustatory circumvallate papillae, *CD36* mRNA levels being down-regulated during the dark period in contrast to *GPR120*, 2°) this change is due to food intake and strictly dependent of the presence of lipids in the diet, 3°) CD36 protein levels are also rapidly but transiently decreased by the food intake, a two-fold drop in CD36 protein levels being found 1 h after refeeding, followed by a progressive return to the pre-prandial values, 4°) this down-regulation, which has a post-transcriptional origin, seems sufficient to alter the spontaneous fat preference, independently to change in the *GPR120* gene expression.

**Conclusions/Significance:**

In contrast to GPR120, CD36 appears to be a food-sensitive lipid sensor in the gustatory circumvallate papillae. Lipid-mediated change in lingual CD36 expression might modulate the motivation for fat during a meal, initially high and then gradually decreasing secondary to the food intake. This short-term lipid-mediated effect is reminiscent of sensory-specific satiety. These findings, which highlight the role played by CD36 in the oro-sensory perception of dietary lipids, raise the possibility of novel pharmacological strategies to modify attraction for fatty foods and decrease obesity risks.

## Introduction

Obesity reaches epidemic proportions worldwide and is a major contributor to the global burden of chronic diseases. Chronic overconsumption of fatty foods contributes to this phenomenon [1]. Rodents and humans display a spontaneous preference for lipid-rich foods. However, the molecular mechanisms underlying this pattern of eating behaviour in mammals remain unclear. The oro-sensory perception of dietary lipids was long thought to involve only textural and olfactory cues. Recent findings challenge this limited viewpoint, strongly suggesting that the sense of taste also plays a significant role in dietary lipid perception and might therefore be involved in the preference for fatty foods and, consequently, in the obesity risk [2].

Compelling evidences implicate the multifunctional protein CD36 as a gustatory lipid sensor. This receptor-like glycoprotein, which belongs to the scavenger receptor family [3], binds saturated and unsaturated long-chain fatty acids (LCFA, number of carbons ≥16) with an affinity in the nanomolar range [4]. CD36 is found in rodent lingual epithelium in which it is strictly restricted to some taste bud cells (TBC) [5], [6]. *CD36* gene inactivation abolishes spontaneous fat preference [6], [7] and the cephalic phase of digestion triggered by a LCFA deposition onto the tongue in the mouse [6]. These physiological effects take place through the gustatory circuitry [8]. Indeed, the spontaneous preference for or, conversely, the conditioned aversion to LCFA require intact gustatory nerves. Moreover, neuronal activation in the gustatory area of the nucleus of the solitary tract elicited by a lingual deposition of LCFA in wild-type mice cannot be reproduced in *CD36*-null animals. Finally, LCFA selectively trigger a rapid and huge increase in [Ca^2+^]_i_ in CD36-positive TBC isolated from mouse circumvallate papillae (CVP). This change, initiated by the phosphorylation of Src protein tyrosine kinases (Src-PTK), leads to the release of neurotransmitters (*i.e.* serotonin and in less extent norepinephrine) which activates the gustatory afferent nerve fibers [9]. Altogether these data strongly highlight the crucial role played by CD36 in the oro-sensory perception of dietary lipids in the mouse. This last finding seems paradoxical since CD36 does not belong to the G protein-coupled receptor (GPCR) family whereas most of the other taste receptors, such as T1Rs and T2Rs responsible for sweet, umami and bitter tastes, do [10]. It has been recently reported that two members of the GPCR family displaying specificity for LCFA also play a role in the taste for fat. GPR40 and GPR120 are specifically expressed in the gustatory epithelium of the tongue in the mouse [11]. Knock-out mice lacking GPR40 or GPR120 have diminished preference for oleic acid and linoleic acid solutions [11]. Contrary to these authors, we have not been able to detect GPR40 mRNA in mouse CVP, similarly to Matsumura and colleagues in the rat [12]. Origin of this discrepancy is unclear. By contrast, we confirm the presence of GPR120 in mouse taste buds which raises the question of the respective role(s) played by CD36 and GPR120 in the coding mechanisms for fat taste at the periphery. In this report, expression of genes encoding for *CD36* and *GPR120* in mouse CVP was explored during the day-night cycle and in mice subjected to nutritional manipulations. Physiological consequences on spontaneous lipid preference were analyzed using behavioural approaches.

## Materials and Methods

### Ethics Statement

French guidelines for the use and the care of laboratory animals were followed and experimental protocols were approved by the animal ethics committee of Burgundy University (approval codes B0110, B0210 and B0610).

### Animals and experimental procedures

Animals were housed in a controlled environment (constant temperature and humidity, darkness from 7 pm to 7 am) and were fed a standard laboratory chow containing 3% of lipids (w/w, soybean oil; Mucedola, Italy). C57Bl/6J wild-type mice were purchased from Charles River Laboratories (France). *CD36* ^−/−^ [13], *CD36* ^+/−^ as well as *CD36* ^+/+^ control littermates were bred locally on a C57Bl/6J background.

#### Diurnal rhythm

Male wild-type mice fed the standard laboratory chow *ad libitum* were used to study gene expression in the CVP. The experiment started at 10 am. Mice were sacrificed every 3 h during 24 h. Animals were anesthetized by an intra-peritoneal injection of ketamine and xylazine mixture (10 mg/100 g of body weight each, in a saline solution). Blood was collected on heparin, plasma recovered by centrifugation (2600 rcf, 10 minutes at 4°C) and conserved at −20°C until biochemical analyses. CVP and surrounding non-gustatory epithelium were isolated for RT-PCR analyses. During the dark phase, experiments were carried-out under red light.

#### Nutritional experiments

Animals were anesthetized by an intra-peritoneal injection of pentobarbital (15 mg/100 g of body weight). In a first experiment, male and female mice were fed a standard diet (3% lipids, w/w). Controls were fasted overnight. In a second experiment, male mice were fed an alipidic (0%) or a hyperlipidic (30%, w/w) diet. Controls were fasted overnight. Diet was reconstituted with soybean oil from a free-fat powder (SAFE, France). In a third experiment, male mice were fasted overnight or fed a low lipidic chow (0.5%, w/w, soybean oil; Mucedola special diet). In other experiments, male mice were fasted overnight and then refed the standard laboratory chow for 1 h, 4 h, 6 h or 11 h. Control group was fasted overnight.

#### Oral deposition of lipids

Male mice were fasted overnight. At t = 0 and t = 15 min, they received a lingual deposition of soybean oil. At t = 60 min, animals were anesthetized by an intra-peritoneal injection of pentobarbital (15 mg/100 g of body weight) and circumvallate papillae were harvested for western blotting analyses.

#### Behavioural experiments

Male *CD36* ^+/+^, *CD36* ^+/−^ and *CD36*-null mice were submitted to double choice tests using contact lickometers (Med Associates, USA). Mice were offered for 30 min a pair of bottles of water and 4% sucrose (Sigma-Aldrich, USA) in experimental cages for 2 days as a training. Since LCFA were added in mineral oil to minimize textural cues, mice were subjected on day 3 to mineral oil (Cooper, France) alone to avoid neophobia. A double choice test between control solution (mineral oil) and experimental solution (mineral oil + different concentrations of linoleic acid; Sigma-Aldrich) was performed on day 4. Mice were food and water deprived 11 h before the test which took place 3 h after the beginning of the dark period. Position of bottles (on the right or the left) was changed daily to avoid the development of side preference. Data were analyzed for 5 min from the first lick and preference for the experimental solutions (ratio between the number of licks on experimental bottle and the total number of licks) was calculated.

### Papillae isolation

Wild-type, *CD36^+/−^* or *CD36^−/−^* mice were sources of taste tissue. Gustatory papillae were isolated according to previously published procedures [6]. In brief, lingual epithelium was separated from connective tissue by enzymatic dissociation (elastase and dispase mixture, 2 mg/ml each in Tyrode buffer: 140 mM NaCl, 5 mM KCl, 10 mM HEPES, 1 mM CaCl2, 10 mM glucose, 1 mM MgCl2, 10 mM Na pyruvate, pH 7.4) and papillae dissected under a microscope. Epithelium surrounding the papillae was also collected to serve as non-sensory control tissue. Samples were snap-frozen in liquid nitrogen and stored at −80°C until RNA extraction or lysed in a buffer for western blot analyses.

### Real-time RT-PCR

Total RNA from gustatory papillae and surrounding non-gustatory epithelium (negative control) were extracted using RNeasy mini-columns (Qiagen, USA). Genomic DNA digestion was performed using the RNase-free DNase Set (Qiagen). First-strand cDNA was generated by reverse transcription from total RNA (Omniscript Reverse Transcription, Qiagen). Levels of mRNA transcripts were determined by real-time RT-PCR (StepOnePlus apparatus, Applied biosystems, USA). α-gustducin, a G protein considered as a specific TBC marker, was systematically assayed to assess the purity of papillae preparations. RNA levels were normalized against levels of 18S ribosomal or 36B4 RNA transcripts in the same samples. Primer probe sets were designed with Primer3 software tool using gene sequences from the GenBank database or purchased directly from Applied Biosystems. PCR amplification was done using Sybrgreen (Power SYBR Green PCR Master Mix, Applied biosystems) or Taqman (Universal Taqman PCR Master Mix, Applied biosystems) technology. The oligonucleotide sequences of primers and probes are shown in Table 1. The comparative 2^ΔΔ^CT method was used for relative quantification [14].

**Table 1:** Sequences and GenBank numbers of primers used for RT-PCR amplifications.

Gene name	Nucleotides sequences (5′→ 3′) or Applied Biosystems Taqman Assay ID details	Pubmed accession number
CD36	Forward: GGCCAAGCTATTGCGACATGProbe: CACAGACGCAGCCTCCTTTCCACCTReverse: CCGAACACAGCGTAGATAGAC	NM_007643
GPR120	Mm01198944_m1	NM_181748
α-gustducin	Forward: ACACATTGCAGTCCATCCTAGCProbe: TGAAGTTGTTCTTGGTCCTCTCGGCTCCReverse: ATCACCATCTTCTAGTGTATTTGCC	XM_144196
18S	Forward: TAAGTCCCTGCCCTTTGTACACAReverse: GATCCGAGGGCCTCACTAAAC	X00686
36B4	Forward: GCCACCTGGAGAACAACCCProbe: AGGTCCTCCTTGGTGAACACGAAGCCReverse: GCCAACAGCATATCCCGAATC	NM_007475

### Western blotting

Samples were homogenized using a micro-potter in a TSE buffer (50 mM Tris HCl, 150 mM NaCl, 1 mM EDTA, 1% nonidet P.40). Protein concentration in homogenates was assayed using a BCA kit (Perkin Elmer, USA). After being separated by SDS-PAGE, proteins were transferred to a PVDF membrane by electroblotting. After being blocked using a TBS buffer containing 5% BSA and 0.05% Tween 20, membranes were incubated overnight at 4°C with a primary antibody (Table 2). After a set of washes, the appropriate peroxidase-conjugated secondary antibody was added. Antibody labeling was detected by chemiluminescence (ECL-plus reagent, Perkin Elmer). GAPDH was used as an internal reference protein.

**Table 2:** Primary antibodies used for Western blotting analyses.

Antigen	Host	Vendor	Dilution
CD36	Goat	R&D Systems	1∶500
GPR120	Rabbit	MBL	1∶500
GAPDH	Mouse	Millipore	1∶500

### Immunohistochemistry

Excised CVP from wild-type or *CD36*-null mice were fixed for 2–3 hours in 4% paraformaldehyde, cryoprotected overnight with 30% sucrose in 0.1 M phosphate buffer (pH 7.4) and then embedded in OCT medium (Tissue-Tek, Sakura Finetek). Cryostat sections (10 µm) were air dried for 2 h at room temperature and then rehydrated in 0.1 M PBS (pH 7.4) for 10 min. Rehydrated sections were incubated during 1 h with PBS containing 0,3% Triton X-100 and then blocked with 10% fatty acid-free BSA in PBS for 40 min. Next, the slices were incubated overnight at 4°C with an anti-GPR120 (1∶500 dilution; MBL, USA) or an anti-α-gustducin (1∶100 dilution; Santa Cruz Biotechnology Inc., USA) primary antibody, both raised in rabbit. Specificity of the GPR120 antibody was assayed in mouse jejunum: only some enteroendocrine cells were positively stained (not shown). Moreover, specificity of this antibody was documented elsewhere [11]. After washing, sections were incubated for 1 h at room temperature with a fluorescent anti-rabbit secondary antibody (Alexa 568 or 488, 1∶1000 dilution; Invitrogen, USA). After washing, slices were counterstained with Hoechst reactive (0.05 mg/ml; Sigma-Aldrich) to stain the nuclei. Slices were then analyzed under an epifluorescence microscope (Zeiss) at the IFR100 Cellular Imaging Platform. In no cases was fluorescent staining observed when the primary antibody was omitted.

### Biochemical analyses

Plasma glucose and triglycerides levels were assayed using specific bioMérieux kits (glucose RTU kit and the TG Pap150 kit, respectively).

### Statistics

Results are expressed as Means ± SEM. The significance of differences between groups was evaluated with SigmaStat (Systat Software, Germany). We first checked that the data for each group were normally distributed and that variances were equal and then carried out ANOVA, two-tailed Student's *t* test or Mann-Whitney tests. A *P* value of less than 0.05 was considered to be statistically significant.

## Results

### CD36 and GPR120 genes display specific diurnal rhythms in mouse circumvallate papillae

**Figure 1:**
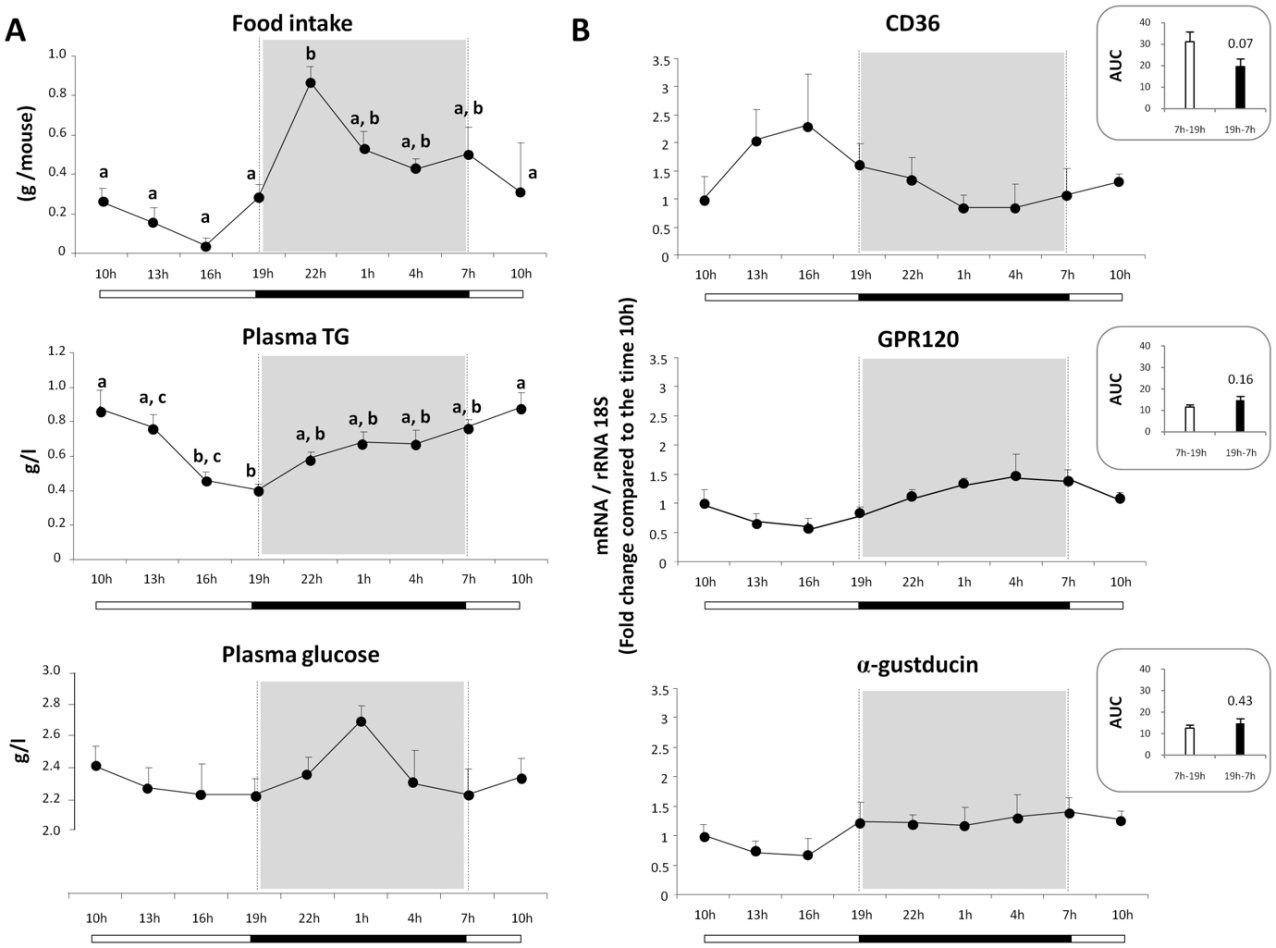
*CD36* and *GPR120* mRNA transcripts display different diurnal rhythms in mouse circumvallate papillae. **A**: Diurnal rhythm of food intake, plasma triglycerides (TG) and glucose levels in mice fed *ad libitum* a standard laboratory chow. Means ± SEM (n = 10). **B**: *CD36*, *GPR120* and *α-gustducin* mRNA levels determined by real-time PCR in circumvallate papillae from mice fed *ad libitum* a standard laboratory chow. Insert: comparison of area under the curve (AUC) between the light and the dark periods. Each value corresponds to a pool of total RNA from 2 mice. Means ± SEM (n = 5).

CD36 or GPR120 gene invalidation deeply affects the spontaneous preference for lipid-enriched solutions in mice subjected to behavioural tests. To better understand the respective function played by these lipid-binding proteins in mouse gustatory papillae, the nycthemeral regulation of *CD36* and *GPR120* gene expression was studied in CVP. The 24-hours cycle is characterized by physiological changes in feeding behaviour, with a peak of food consumption occuring during the dark period associated with changes in various plasma parameters including plasma triglycerides and glucose levels (Fig. 1-A). *CD36* mRNA levels in CVP tended to be lower during the dark period than during the light period (Fig. 1-B). By contrast, *GPR120* mRNA levels did not show a similar change along a day suggesting that *CD36* and *GPR120* genes display different diurnal rhythms. Comparison of areas under the curve (AUC) is consistent with this assumption (Fig. 1-B). mRNA levels of the G protein α-gustducin, known to be involved in the transduction of sweet, bitter and *umami* tastes and used here as a specific CVP marker, remained grossly stable along a day (Fig. 1-B).

### CD36 and GPR120 gene expression are differentially regulated by dietary lipids in circumvallate papillae

**Figure 2:**
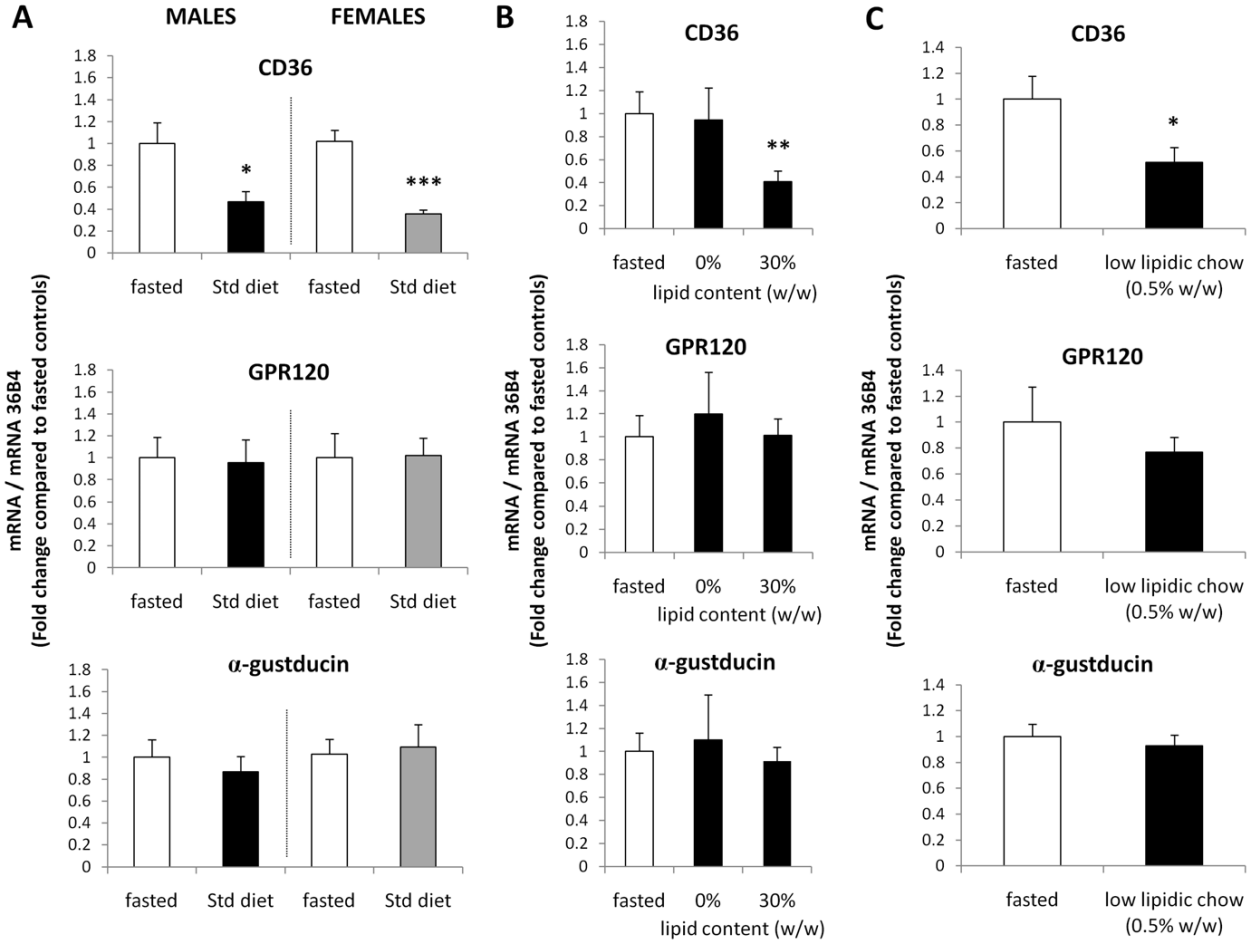
CD36 and GPR120 gene expression are differentially regulated by dietary lipids in mouse circumvallate papillae. **A**: *CD36*, *GPR120* and *α-gustducin* mRNA levels determined by real-time PCR in circumvallate papillae from male and female mice fed *ad libitum* a standard (Std) diet (3% lipids, w/w). Controls were fasted overnight. Each value corresponds to a pool of total RNA from 2 mice. Means ± SEM (n = 6). * p<0.05; *** p<0.001. **B**: *CD36*, *GPR120* and *α-gustducin* mRNA levels determined by real-time PCR in circumvallate papillae from male mice fed *ad libitum* an alipidic (0%) or a hyperlipidic (30% lipids, w/w) diet. Controls were fasted overnight. Each value corresponds to a pool of total RNA from 2 mice. Means ± SEM (n = 4–6). ** p<0.01. **C**: *CD36*, *GPR120* and *α-gustducin* mRNA levels determined by real-time PCR in circumvallate papillae from male mice fed *ad libitum* a low lipidic chow (0.5% lipids, w/w). Controls were fasted overnight. Each value corresponds to a pool of total RNA from 2 mice. Means ± SEM (n = 4). * p<0.05.

To explore whether this differential regulation of *CD36 and GPR120* genes was related to the nutritional status of mice, *CD36* and GPR120 mRNA levels were assayed in CVP from fasted or fed mice. *CD36* mRNA levels were systematically lower in fed mice than in fasted animals. This nutritional regulation was not gender-dependent since it is retrieved in males and females. In contrast, *GPR120* or *α-gustducin* mRNA levels remained unchanged (Fig. 2-A). To determine whether the lipid content of diet was responsible of the down-regulation of *CD36* gene, levels of mRNA encoding for *CD36* and *GPR120* were assayed in CVP from mice fed an alipidic diet (0% lipid) or a high fat diet (30% lipid, w/w) and compared to the fasted controls (Fig. 2-B). Interestingly, the decrease in *CD36* mRNA found in mice subjected to the high fat diet was not reproduced with the alipidic diet demonstrating the lipid-dependent origin of this nutritional regulation. The fact that *GPR120* mRNA levels remained unchanged in these conditions suggests that this gene is insensitive to the lipid content of diet in contrast to CD36 (Fig. 2-B). To further explore the sensitivity to lipid of the *CD36* gene, mice were fed a diet especially low in fat (0.5%, w/w). As shown in the Fig. 2-C, this minute quantity of fat was sufficient to trigger a two-fold decrease in *CD36* mRNA levels as compared to fasted controls. As expected, *GPR120* and *α-gustducin* gene expression remained unchanged in this condition.

### There is a lipid-dependent drop in CD36 protein levels in circumvallate papillae while the amount of GPR120 protein remains stable

**Figure 3:**
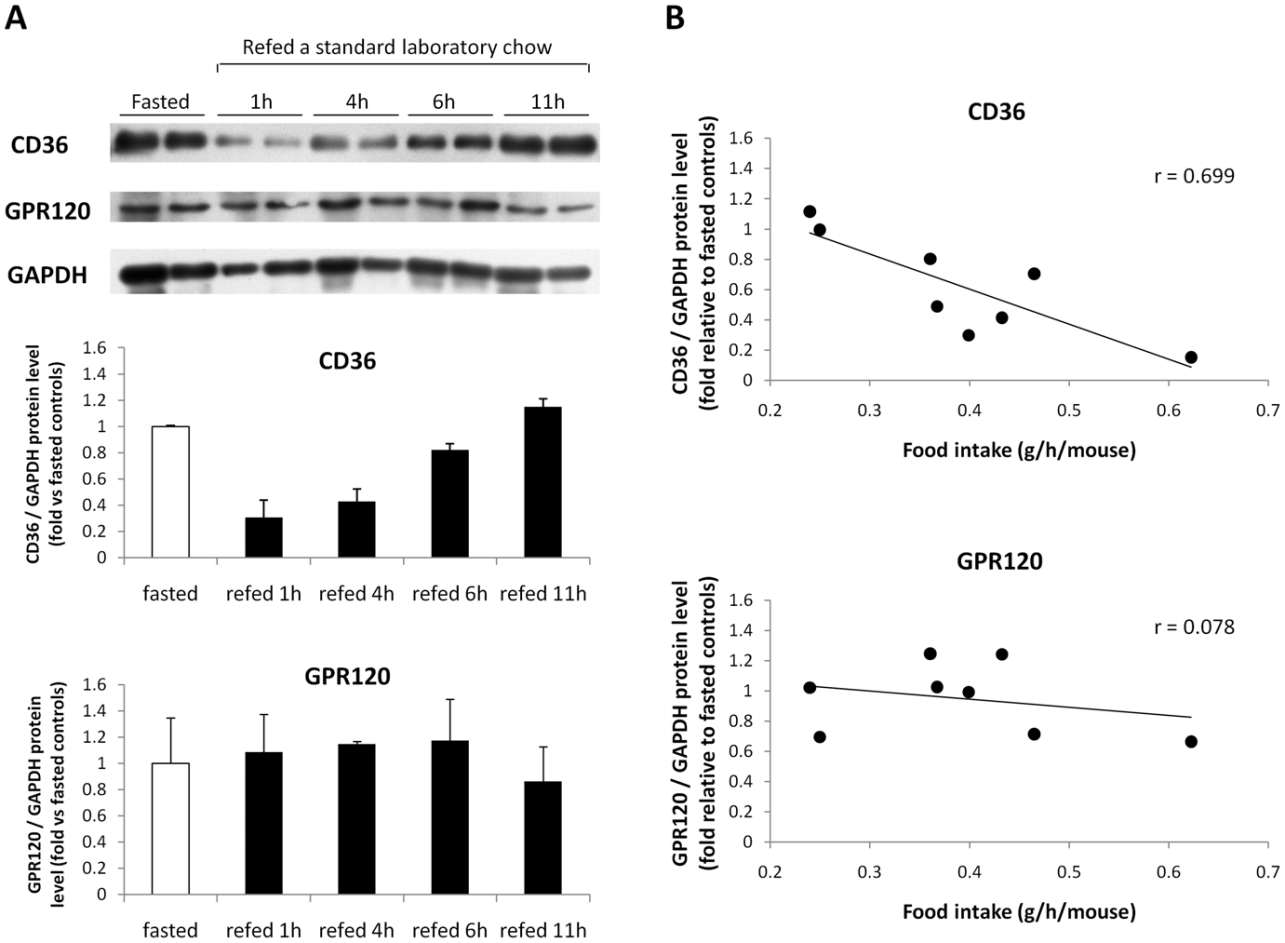
*CD36* and *GPR120* proteins in circumvallate papillae are differentially regulated during fasting/refeeding sequences. **A**: CD36 and GPR120 protein levels determined by western blotting in circumvallate papillae from fasted controls or mice refed *ad libitum* a standard laboratory chow for 1 h, 4 h, 6 h or 11 h. Each point corresponds to a pool of total proteins from 4 mice (n = 2). The mean CD36/GAPDH and GPR120/GAPDH protein ratios were also analyzed. Error bars are SEM. **B**: Relationship between CD36 or GPR120 protein levels and food intake.

**Figure 4:**
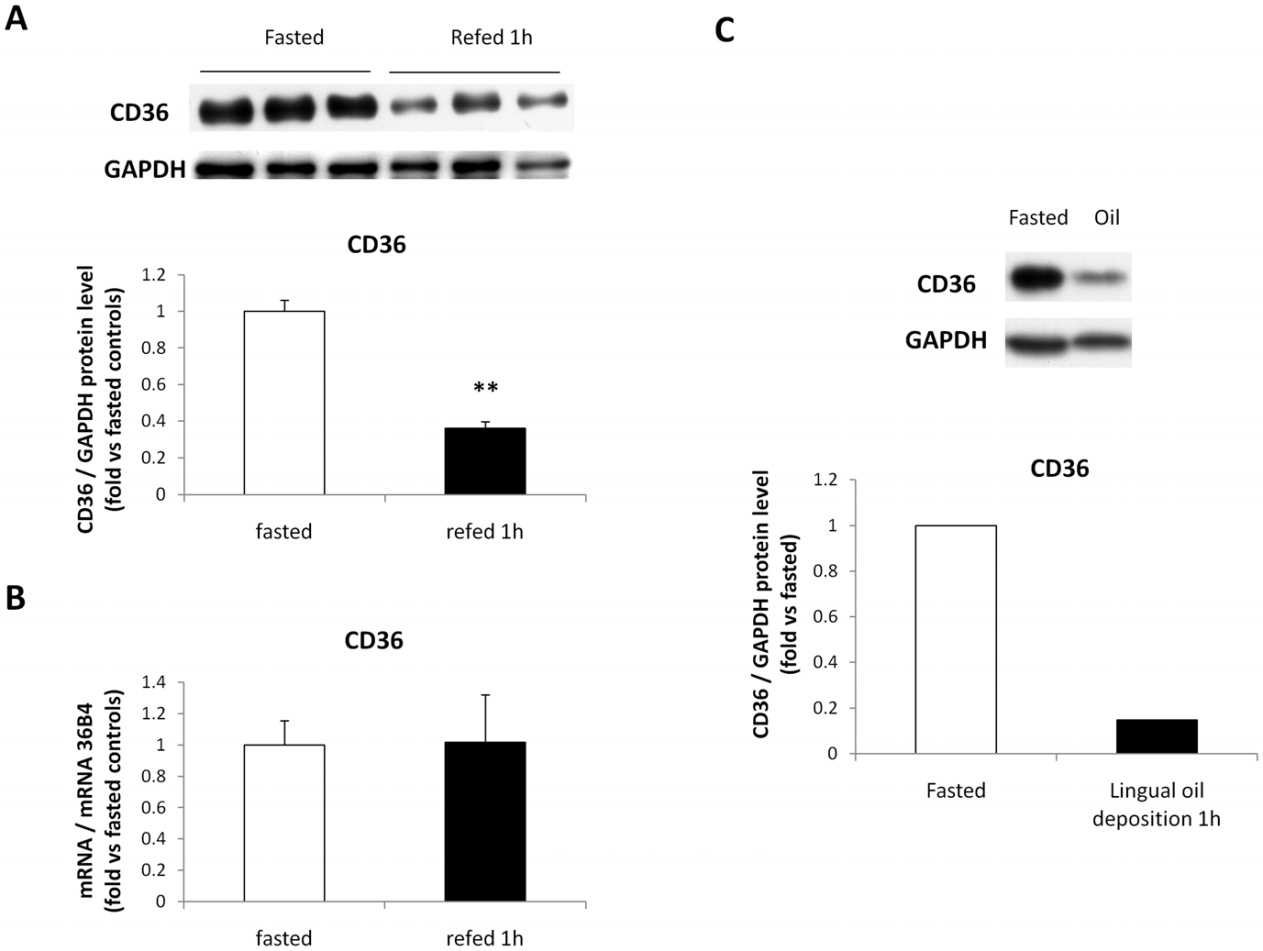
Dietary lipids induce a direct post-transcriptional down-regulation of lingual CD36. **A**: CD36 protein levels determined by western blotting in circumvallate papillae from fasted controls or mice refed *ad libitum* a standard laboratory chow for 1 h. Each point corresponds to a pool of total proteins from 3 mice. Means ± SEM (n = 3). ** p<0.01. **B**: *CD36* mRNA levels determined by real-time PCR in circumvallate papillae from fasted controls or mice refed *ad libitum* a standard laboratory chow for 1 h. Each value corresponds to a pool of total RNA from 2 mice. Means ± SEM (n = 5–6). **C**: CD36 protein level determined by western blotting in circumvallate papillae from fasted mice 1 h after a lingual deposition of soybean oil at t = 0 and t = 15 min. Controls were fasted mice. Each point corresponds to a pool of total proteins from 3 mice. Data are representative of two different experiments.

Time-course experiment was performed by Western blotting to explore whether CD36 and GPR120 protein levels in CVP were also affected by the food intake during a fasting/re-feeding sequence. A dramatic drop in CD36 protein levels was found 1 hour after re-feeding, followed by a progressive return to levels observed during fasting (Fig. 3-A). An inverse correlation between food consumption and CD36 protein levels was found in CVP (Fig. 3-B). In contrast, no food-mediated regulation of GPR120 protein levels was observed (Fig. 3A & B-). To further explore feeding-induced down-regulation of CD36 in CVP, *CD36* gene expression was compared in fasted and 1 h refed mice. Consistent with the time-course experiment (Fig. 3-A), a 3-fold decrease in CD36 protein levels was found in mice refed during 1 h (Fig. 4-A). Surprisingly, no change in *CD36* mRNA levels occurred in these conditions suggesting that the drop of CD36 protein has a post-transcriptional origin (Fig. 4-B). Decrease in CD36 protein levels in CVP was efficiently reproduced when oil was directly deposited onto the tongue in fasted mice excluding involvement of post-ingestive influences in this regulation (Fig. 4-C).

### The lipid-mediated down-regulation of lingual CD36 gene should be sufficient to affect the preference for lipids independently to GPR120 expression

**Figure 5:**
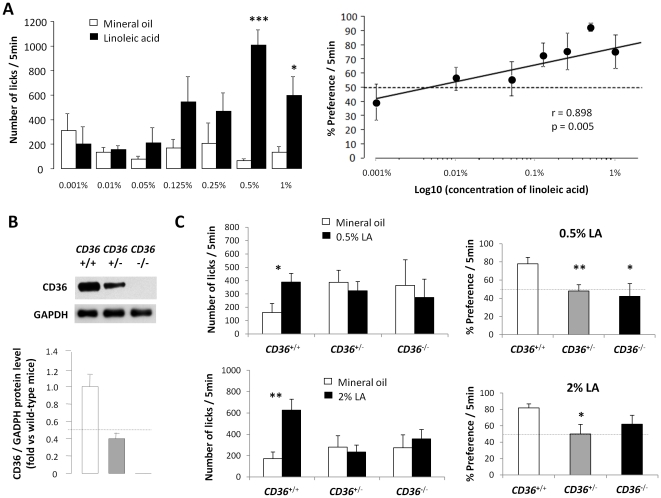
CD36 protein levels in circumvallate papillae can affect lipid preference. **A**: Number of licks of wild-type mice during 5 min behavioural experiments (*i.e.* two-bottle preference tests) as a function of concentrations of linoleic acid (LA) in mineral oil used to minimize textural cues. Means ± SEM (n = 4–9). * p<0.05; *** p<0.001. Relationship between the percentage of preference and the logarithm of linoleic acid concentration is also shown. Dotted line represents a lack of preference. **B**: CD36 protein levels determined by western blotting in circumvallate papillae of *CD36* ^+/+^, *CD36* ^+/−^ or *CD36* ^−/−^ mice. Each point corresponds to a pool of total proteins from 4 mice (n = 2). Error bars are SEM. A representative immunoblot of CD36 and GAPDH is shown. **C**: Number of licks and percentage of preference for linoleic acid (LA) during 5 min behavioural experiments in *CD36* ^+/+^, *CD36* ^+/−^ or *CD36* ^−/−^ mice. Animals were allowed access to a control solution (mineral oil) and an experimental one (mineral oil + 0.5% or 2% LA). Dotted line represents a lack of preference. Means ± SEM (n = 6–11). * p<0.05; ** p<0.01.

**Figure 6:**
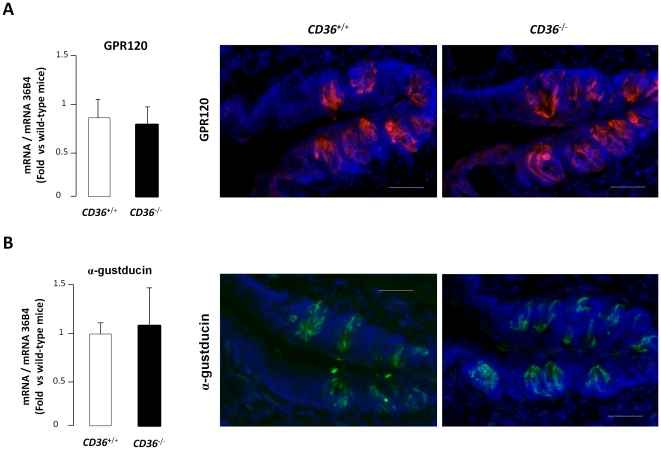
*GPR120* gene expression is unaffected by *CD36* gene invalidation. **A**: *GPR120* mRNA levels determined by real-time PCR in circumvallate papillae of *CD36* ^+/+^ or *CD36* ^−/−^ mice. Each value corresponds to a pool of total RNA from 3 mice. Means ± SEM (n = 4). Immunodetection of GPR120 protein (red) in circumvallate papillae of *CD36* ^+/+^ or *CD36* ^−/−^ mice. Hoechst (blue) was used to stain the nuclei. Scale bar is 50 µm. **B**: *α-gustducin* mRNA levels determined by real-time PCR in circumvallate papillae of *CD36* ^+/+^ or *CD36* ^−/−^ mice. Each value corresponds to a pool of total RNA from 3 mice. Means ± SEM (n = 4). Immunodetection of α-gustducin protein (green) in circumvallate papillae of *CD36* ^+/+^ or *CD36* ^−/−^ mice. Hoechst (blue) was used to stain the nuclei. Scale bar is 50 µm.

If CD36 plays a significant role in the chemodetection of LCFA in the oral cavity, its nutritional-mediated down-regulation in gustatory papillae might affect the preference for lipids during a meal. Unfortunately, by reason of deep changes both in endocrine status and autonomic nervous system activity which might also affect the preference for fat, the comparison of feeding behaviour in fed and fasted mice would not allow to fully answer to the question. To overpass for this limitation, two-bottle preference tests were performed in *CD36* ^+/+^, *CD36* ^+/−^ and *CD36* ^−/−^ mice in which CD36 levels in circumvallate papillae are genetically established (Fig. 5). As in our previous long-term two-bottle preference tests [6], linoleic acid (LA) was used. To minimize textural cues, post-ingestive effects and lipid-mediated activation of CD36 in other tissues, control and experimental solutions containing LA were prepared using mineral oil (saturated long-chain hydrocarbon) and behavioural tests were analyzed for 5 min from the first lick using a computer-controlled lickometer. Experiments were performed using 0.5% LA since this concentration led to an optimal behavioural response in wild-type mice with this protocol (Fig. 5-A). Surprisingly in these conditions, LA preference found in wild-type animals was fully abolished not only in *CD36*-null mice but also in heterozygous *CD36* ^+/−^ animals in which CD36 protein expression is two-fold lower in CVP (Fig. 5-B & C). Similar data were also found when *CD36* ^+/−^ mice were subjected to a 4-fold higher LA concentration (*i.e.* 2%) suggesting that, like homozygous mice, heterozygous mice are unable to detect large quantity of free LCFA in a textured solution (Fig. 5-C). To take into consideration the putative role played by GPR120 in this loss of fat detection, impact of *CD36* gene invalidation on *GPR120* gene expression was studied in mouse CVP. Levels of *GPR120* mRNA and protein localization in taste buds remained similar in wild-type and *CD36*-null mice CVP. (Fig. 6-A). The G protein α-gustducin was used as a taste bud cell marker (Fig. 6-B).

## Discussion

Many recent studies strongly suggest the existence of a specific gustatory system devoted to the detection of LCFA in rodents and, likely, in humans [2], [15]. Two unrelated lipid-sensor candidates, the multifunctional glycoprotein CD36 and the G protein-coupled receptor GPR120, displaying similar binding specificities for LCFA [4], [16], have been identified in gustatory papillae in the mouse. Inactivation of the gene encoding for *CD36* [6], [7] or *GPR120* [11] renders mice unable to properly detect the presence of LCFA in a textured solution during behavioural tests. This effect seems to be specific since the disruption of the *CD36* or *GPR120* genes does not alter the architecture of taste buds [6], [11] and sweet preference or bitter aversion are maintained in *CD36* ^−/−^ and *GPR120* ^−/−^ mice [6], [11]. If CD36-mediated signaling in taste buds is partially deciphered [9], mechanisms by which GPR120 affects fat preference are not yet established. Collectively, these findings raise the question of the respective role(s) of CD36 and GPR120 in gustatory papillae in the mouse.

We report herein that the expression of the *CD36* gene in mouse CVP is subjected to a short-term lipid-mediated down-regulation during food intake in contrast to what is found for the *GPR120* gene. The physiological relevance of this change is supported by its diurnal rhythm. Indeed, a slight decrease in *CD36* mRNA levels occurred during the dark period characterized by a sustained food intake while GPR120 mRNA remained unchanged. The fact that this decrease is reproduced when fasted mice are fed or refed a standard laboratory chow suggests its nutritional origin. It is a very sensitive regulation strictly dependent of the presence of lipid in diet since it is still retrieved in mice refed a diet containing only 0.5% (w/w) lipids but lacking when animals were subjected to an alipidic diet. This observation correlates quite well with the high binding affinity of CD36 for LCFA [4]. Interestingly, direct oil deposition onto the tongue is sufficient to trigger the decrease of CD36 protein in CVP suggesting a local regulation independent from subsequent post-oral influences. Moreover, the post-transcriptional origin of this regulation is likely since the decrease in the CD36 protein in CVP took place while CD36 mRNA remained unchanged. Interestingly, we have recently identified a similar nutritional-mediated decrease of *CD36* gene expression in the small intestine in refed rats or oil force-fed mice. In this tissue, the down-regulation of *CD36* gene expression was consecutive to a lipid-mediated disappearance of CD36 protein from brush bordure membrane of enterocytes followed by a rapid degradation by the ubiquitin-proteasome pathway [17]. This event triggered the activation of a signaling cascade involving the ERK1/2 pathway leading to an optimization of the chylomicron formation [17]. In the present study, the lipid-mediated decrease in CD36 protein levels being found using total CVP homogenates, it can be thought that it also results from a CD36 degradation. As reported for numerous surface receptors, such a negative regulatory feedback might elicit a physiological desensitization of lingual CD36 secondary to a persistent exposure to dietary lipids.

Lipid-induced modulation of CD36 in CVP constitutes an additive feature consistent with its involvement in the oro-sensory perception of dietary lipids. Indeed, it has been previously shown that CD36 expression is strictly restricted to the apical side of some TBC lining the pore of gustatory papillae in the lingual epithelium from rat [5], [6] and human [18]. Moreover, it exhibits a receptor-like structure, with an extracellular binding pocket [19] and a C-terminal cytoplasmic tail able to interact with kinases [20] constituting a functional complex able to activate cell signaling in TBC in a lipid-dependent manner. Consistent with this assumption, experiments using CD36 positive TBC isolated by immuno-magnetism from mouse CVP have shown that activation of CD36 by LCFA leads to the recruitment and activation of Src-PTK [9]. This event triggered a prompt and huge rise in intracellular ionized calcium levels ([Ca^2+^]_i_) [8] leading to the release of serotonin and nor-epinephrin, neuro-transmitters known to activate afferent gustatory nerve fibers [9]. It is noteworthy that all steps of this cascade are strictly CD36-dependent since they are not reproduced neither in CD36-negative TBC nor in CD36-positive TBC pre-treated with the pharmacological CD36 binding inhibitor, sulfo-N-succinimidyl oleic acid ester (SSO) [9]. Finally LCFA deposition onto the tongue induces the neuronal activation in the nucleus of solitary tract (NST) areas in the brainstem known to receive afferent fibers from gustatory nerves (*i.e.* chorda tympani and glossopharyngeal nerves). Interestingly, this activation appears to be CD36-dependent, since it is not reproduced in *CD36*-null mice subjected to an oral LCFA stimulation [8].

From a physiological point of view, we confirm in the present report the crucial role played by lingual CD36 in the oro-sensory detection of LCFA using a short-term (5 min) two-bottle preference test controlled by a computer. In these conditions, which minimize post-ingestive influences and CD36 activation in other tissues, *CD36*-null mice but not wild-type one are unable to discriminate the solution containing LCFA compared to the control solution. Interestingly, this lack of detection is also found in heterozygous *CD36* ^+/−^ animals, expressing only half of the CD36 protein in CVP. Therefore, it is tempting to speculate that the two-fold drop in CD36 protein levels elicited by the refeeding in presence of dietary lipids might be sufficient to decrease the preference for lipids. According to this assumption, it can be thought that the lipid-mediated change in *CD36* gene expression in CVP might modulate the motivation for fat during a meal, initially high and then gradually decreasing during the food intake. This physiological negative feed-back is reminiscent of sensory-specific satiety which refers to a temporary decline in pleasure derived from consuming a certain food in comparison to other unconsumed foods [21]. It is noteworthy that such an effect seems to be independent to *GPR120* since its gene expression is unaffected by the food intake or the CD36 gene ablation.

The lipid-mediated down-regulation of CD36 protein found both in taste buds and intestinal mucosa also suggests that CD36 might play a similar role throughout the oro-intestinal tract, as a lipid sensor [22]. This assumption is supported by recent data showing that digestive and absorptive processing of ingested food is coordinated by specialized nutrient sensing systems in the intestinal tract similar to what is found in the oral cavity. For instance, the same T1R3 sweet taste receptor, that senses sugar in the mouth, also detects sugar in the intestinal lumen and triggers physiological responses affecting sugar metabolism [23], [24]. These observations suggest the existence of a “*functional continuum*” throughout the oro-intestinal tract responsible for the real-time analysis and control of ingested energy nutrients and their subsequent digestion, absorption and metabolic fate in the body.

Compelling evidences show that oral perception of LCFA affects food intake contributing to the risk of weight gain and obesity. An inverse correlation between peripheral gustatory sensitivity to poly-unsaturated fatty acids and preference for lipid-rich foods was reported in rats [25]. Recently, experiments performed in healthy humans have highlighted the relationship between the oro-sensory fat perception and the body mass index. Hypersensitivity to lipids was associated with lower energy consumption, fat intake and body mass index [26]. Whether threshold of sensitivity to dietary lipids is dependent of CD36 and/or GPR120 in humans remains to be determined. A better understanding of molecular mechanisms responsible for the oro-sensory reception of dietary lipids and their physiological impacts on the feeding behaviour should allow the development of new therapeutic and nutritional strategies for mitigating excess food intake.
